# Small nucleolar RNA expression profiles: A potential prognostic biomarker for non-viral Hepatocellular carcinoma

**DOI:** 10.1016/j.ncrna.2024.06.009

**Published:** 2024-06-12

**Authors:** Venkata Ramana Mallela, Phanindra Babu Kasi, Dattatrya Shetti, Andriy Trailin, Lenka Cervenkova, Richard Palek, Ondřej Daum, Vaclav Liska, Kari Hemminki, Filip Ambrozkiewicz

**Affiliations:** a Laboratory of Translational Cancer Genomics, Biomedical Center, Faculty of Medicine in Pilsen, Charles University, Alej Svobody 1665/76, 323 00, Pilsen, Czech Republic; b Laboratory of Cancer Treatment and Tissue Regeneration, Biomedical Center, Faculty of Medicine in Pilsen, Charles University, Alej Svobody 1665/76, 323 00, Pilsen, Czech Republic; c Department of Surgery, University Hospital in Pilsen and Faculty of Medicine in Pilsen, Charles University, Alej Svobody 80, 323 00, Pilsen, Czech Republic; d Department of Cancer Epidemiology, German Cancer Research Center, Im Neuenheimer Feld 280, 69120, Heidelberg, Germany; e Sikl's Institute of Pathology, Faculty of Medicine and Teaching Hospital in Pilsen, Charles University, Ul. Dr. E. Beneše 13, 30599, Pilsen, Czech Republic; f Bioptická Laboratoř S.r.o., Mikulášské Nám. 4, 32600, Pilsen, Czech Republic; g Department of Integrative Medical Biology, Umeå University, SE-901 87, Umeå, Sweden

**Keywords:** Small nucleolar RNA, Hepatocellular carcinoma, Prognostic marker, Non-viral HCC snoRA47 and snoRD126

## Abstract

Hepatocellular carcinoma (HCC) is a challenging cancer with high mortality rates, limited predictability, and a lack of effective prognostic indicators. The relationship between small nucleolar RNAs (snoRNAs) and HCC is poorly understood. Based on the literature data, snoRNA studies were primarily focused on viral-related causes of HCC, such as Hepatitis B or C viruses (HBV or HCV). According to these studies, we selected four snoRNAs (snoRA12, snoRA47, snoRA80E, and snoRD126) for exploration in the context of non-viral-related causes, including non-alcoholic steatohepatitis (NASH), non-alcoholic fatty liver diseases (NAFLD), and alcohol steatohepatitis. The primary goal of this study was to gain a deeper understanding of how snoRNA expression affects patient outcomes and whether it can serve as a prognostic tool for non-viral HCC. We conducted a study on tissue samples from 35 HCC patients who had undergone resection at Pilsen University Hospital. SnoRA12, snoRA47, snoRA80E, and snoRD126 were studied by quantitative real-time PCR (qRT-PCR) in tumor and non-tumor adjacent tissue (NTAT) samples. Kaplan-Meier analysis was performed to assess the association of snoRNAs expression levels with patient outcomes: time to recurrence (TTR), disease-free survival (DFS) and overall survival (OS). In tumor tissues, snoRA12, snoRA47 and snoRA80E were upregulated, while snoRD-126 was downregulated compared to NTAT. Low expression of snoRA47 and snoRD126 in patients was associated with longer TTR and DFS. The individual expression of snoRA12 and snoRA80E did not show associations with TTR and DFS. However, a combination of medium expression of snoRD126 and snoRA80E was associated with longer TTR and DFS, while high and low expressions of the combined snoRA126 and snoRA80E showed no significant association with TTR, DFS, and OS. Conversely, a combination of high expression of snoRA12 and snoRD126 was associated with shorter TTR. In conclusion, the results indicate that snoRA47 and snoRD126 exhibit good prognostic power specifically for non-viral related HCC. Both snoRA47 and snoRD126 showed favorable prognostication in single and combined analysis when assessing patient outcomes. Also, in combination analysis, snoRA80E and snoRA12 showed favorable prognosis, but not alone.

## 1 Introduction

Hepatocellular carcinoma (HCC) ranks as the third leading cause of cancer-related mortality and is among the six most prevalent cancers. In 2020, almost 906,000 individuals were diagnosed with HCC, with related mortality affecting around 830,200 [[1], [2], [3]]. Non-alcoholic fatty liver disease (NAFLD) is the primary cause of chronic liver disease, ranging from simple steatosis to the more severe non-alcoholic steatohepatitis (NASH). In advanced stages, it can lead to cirrhosis and eventually progress to HCC [4,5]. Additionally, HBV or HCV infections, along with alcohol consumption, significantly impact hepatitis outcomes [6]. Early-stage HCC often presents with minimal or no symptoms, resulting in late detection and limited options for surgical intervention. Given the scarcity of effective therapies for cases deemed inoperable, the identification of new tissue biomarkers becomes crucial. These can play a pivotal role in early detection, enabling improved treatment strategies for liver cancer. Early identification can enhance the prospects for successful intervention and management of HCC [7]. Indeed, curative options such as surgical resection or liver transplantation are available for HCC. However, the eligibility for these curative treatments is limited, with less than 40 % of patients qualifying [8].

SnoRNAs are a family of non-coding RNAs (ncRNAs), typically 60 to 300 nucleotides long, that act as guides within small nucleolar ribonucleoproteins (snoRNPs), which are crucial in modifying and maturing ribosomal RNAs (rRNAs) after transcription [9]. SnoRNAs primarily originate from intronic regions of genes, both protein-coding and non-protein-coding genes. They are broadly categorized into three groups: H/ACA box snoRNAs, C/D box snoRNAs, and small Cajal RNAs (scaRNAs) [10]. Recent evidence increasingly indicates the involvement of various ncRNAs in HCC carcinogenesis, encompassing microRNA (miRNA), long non-coding RNA (lncRNA), and snoRNA [11]. Previous studies have demonstrated the involvement of snoRNAs in the development of various cancers including colorectal cancer (CRC) and HCC [12,13]. SnoRA12 is a C/D box snoRNA that guides 2′-*O*-methylation via conserved C (UGAUGA) and D (CUGA) box motifs, which are common in this snoRNA family [14].

SnoRA12 demonstrated significant dysregulation in lung cancer tissue, displaying high diagnostic performance with an AUC of 0.75, sensitivity of 75 %, and specificity of 70 % [15]. SnoRA80E exhibits strong carcinogenic activity in CRC and HCC. Elevated levels of snoRA80E in tumor tissues correlate with poor prognosis, particularly in HCC [13]. In a study involving 60 pairs of HCC tumor and NTAT, snoRA47 exhibited higher expression levels in tumors. Its correlation with intrahepatic metastasis and TNM stage suggests snoRA47's potential as a promising tissue biomarker for HCC [16]. Ding Y et al. showed that snoRA71A expression was downregulated in 132 HCC tissues compared to NTAT. Low snoRA71A expression was associated with reduced overall survival (OS), this downregulation served as a prognostic biomarker for HCC [17]. Xu et al. research, encompassing 68 tumor and NTAT samples, identified snoRD126 as a promising prognostic marker and potential therapeutic target for HCC. Their findings were validated through serum samples as well as functional studies [18].

In our current study, the primary objective was to understand the expression levels of four distinct snoRNAs (snoRA12, snoRA47, snoRA80E, and snoRD126) in non-viral HCC. These snoRNAs have not been extensively studied in this specific context. Therefore, we aimed to fill this gap in information by investigating their expression levels in non-viral cases.

## 2 Material and methods

### 2.1 Study design

**Fig. 1:**
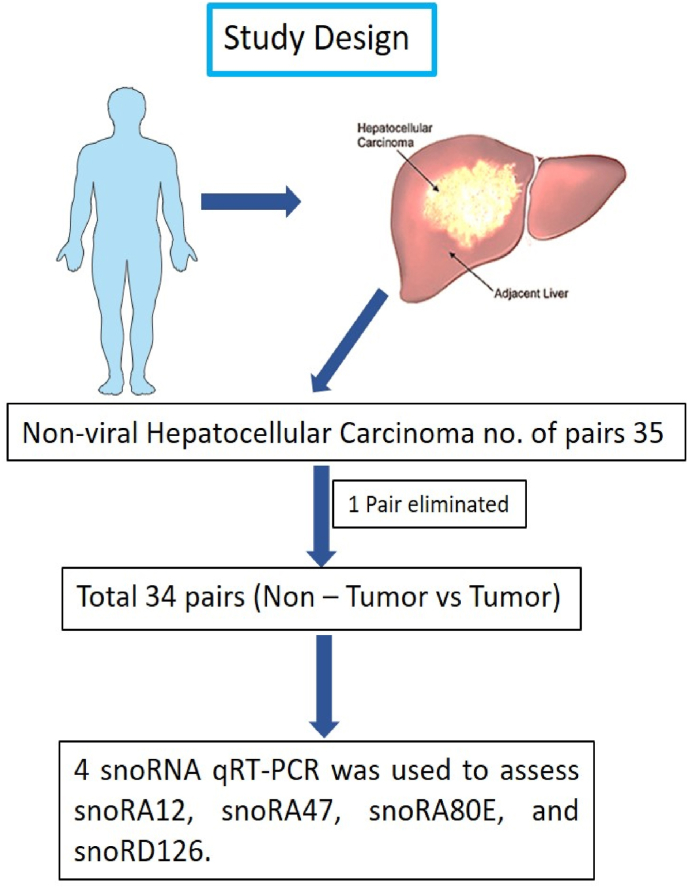
Illustrates the comprehensive workflow implemented in this study, guiding the progression of experimental procedures from sample collection to data analysis. One pair was eliminated due to discrepant data.

The expression of four snoRNA candidates was evaluated in 35 HCC tissues and their respective NTAT samples. The expression profiles were assessed using qRT-PCR and the 2^^ΔΔCt^ method. The study design is illustrated in (Fig. 1). All clinical and pathological data summarized in (Table 1) shown below.

**Table 1:** Demographic and clinical and pathology characteristics of patients.

N.o Patients		
**Age y (Median, min-max)**	71 (29–86)	
**Sex (n=34) n%**	Male	27 (79.4 %)
	Female	7 (20.6 %)
**Patient etiology (n=34) n%**	NAFLD	2 (5.9 %)
	Non-alcoholic steatohepatitis	13 (38.2 %)
	Alcoholic steatohepatitis	6 (17.7 %)
	Cryptogenic chronic hepatitis	5 (14.7 %)
	Cirrhosis	5 (14.7 %)
	Unknown	3 (8.8 %)
**Tumor size cm (n=34) n%**	<5 cm	16 (47 %)
	>5 cm	18 (53 %)
**Tumor grade WHO (n=34) n%**	1	14 (41.17 %)
	2	19 (55.89 %)
	3	1 (2.94 %)
**Growth pattern (n=34) n%**	Desmoplastic	10 (29.4 %)
	Infiltrative	0 (0.0 %)
	Pushing	0 (0.0 %)
	Mixed	9 (26.5 %)
	Unknown	15 (44.1 %)
**Grade of fibrous encapsulation (n=34) n%**	1	3 (8.9 %)
	2	16 (47 %)
	3	15 (44.1)
**Architectural grade (n=34) n%**	1	7 (20.6 %)
	2	6 (17.6 %)
	3	17 (50 %)
	4	4 (11.8 %)
**Amount of stroma within tumor (n=34) n%**	Low	22 (64.7 %)
	High	12 (35.3 %)
**Nuclear grade (n=34) n%**	1	11 (32.3 %)
	2	14 (41.2 %)
	3	9 (26.5 %)
**Nucleolar grade (n=34) n%**	1	9 (26.5 %)
	2	11 (32.3 %)
	3	14 (41.2 %)
**Microvascular invasion (n=34) n%**	Absence	24 (71 %)
	Presence	9 (26 %)
	Unknown	1 (3 %)
**Micronodularity (n=34) n%**	Absence	26 (76.5 %)
	Presence	8 (23.5)
**Micro/macrosatellites (n=34) n%**	Absence	15 (44.1 %)
	Presence	16 (47.1 %)
	Unknown	3 (8.8 %)
**TNM rank (n=34) n%**	2	20 (58.8 %)
	3	9 (26.5 %)
	4	5 (14.7 %)
**AFP IU/ml (Median, min - max) (n=24)**^a^	(3, 1–1133.7)	
**Diabetes (n=34) n%**	Absence	16 (47 %)
	Presence	18 (53 %)

a Missing data.

### 2.2 Patient characteristics

A total of 35 paired (tumor and NTAT) formalin-fixed paraffin-embedded (FFPE) samples of HCC were collected. One pair eliminated discrepant data. All patients underwent primary tumor resection without any neoadjuvant therapy at the University Hospital in Pilsen. All patients gave informed consent and the study design was approved by the Ethical Committee.

### 2.3 RNA isolation

Total RNA was isolated using the RecoverAll™ Total Nucleic Acid Isolation Kit for FFPE (Cat. No. AM197, Thermo Fisher Scientific) following the manufacturer protocol.

### 2.4 RT-PCR and qPCR

The isolated total RNA (4 ng) was reverse transcribed into complementary DNA (cDNA) using the Applied Biosystems™ TaqMan MicroRNA Reverse Transcription Kit. Thermo Fisher Scientific, Inc.) according the manufacturer's protocol, resulting in a final volume of 15 μl. The reverse transcription process consisted of incubation at 16 °C for 30 min, followed by 42 °C for another 30 min. The reaction was then stopped by heating to 85 °C for 5 min, and finally, the samples were held at 4 °C indefinitely.

Relative gene expression was evaluated through qPCR, using 1.33 μl of cDNA per sample in a 20 μl reaction, along with TaqMan ™ Universal Master Mix II no UNG 10 μl, custom TaqMan ^R^ small RNA assays 1 μl, and Nuclease free water 7,67 μl. Ct values were quantified using the Bio-Rad CFX96 qPCR Real-Time PCR Module with the C1000 Touch Thermal Cycler. The frequently employed endogenous control for standardizing the RNA amount in each sample was the miR-16. The cDNA samples underwent amplification in 96-well plates with an initial enzyme inactivation step of 10 min at 95 °C, followed by 40 cycles of denaturation for 15 s at 95 °C, and annealing or extension for 60 s at 60 °C. Expression levels were calculated using the 2−^ΔΔCt^ formula.

### 2.5 Statistical analysis

The collected data were processed using Prism GraphPad statistical software version 8.0.1. Associations between snoRNAs and clinical or pathological data were evaluated using nonparametric Spearman correlation for continuous and ordinal data. For binary variables, we utilized the Mann-Whitney *U* test. Additionally, the Kruskal-Wallis test was employed to analyze the association with growth pattern. For survival analysis, Kaplan-Meier method and log-rank test were employed. The median normalized expression of each snoRNA was chosen as the cut-off value. TTR was defined as the time from the date of tumor resection to the date of diagnosis of recurrence/metastasis. If recurrence was not diagnosed, patients were censored at either the date of death or the date of last follow-up. The appropriate proportion of patients without recurrence was denoted as the recurrence-free proportion. DFS was considered as the time from tumor resection to the date of diagnosis of recurrence/metastasis or death due to any cause. OS was determined as the time from tumor resection to death due to any cause. Statistical significance was determined as p < 0.05. Receiver Operating Characteristic (ROC) Analysis: ROC analysis was conducted for each snoRNA separately for non-tumor adjacent tissues and tumor tissues. ROC analysis is used to evaluate the diagnostic performance of a biomarker and determine the optimal cut-off values. A comprehensive analysis was conducted involving 17 clinical and pathological variables. Among these, 5 were clinical variables (age, gender, tumor size, TNM stage, and AFP concentration), while 12 were pathological variables (such as tumor grade), growth pattern, grade of fibrous incapsulation, architectural grade, stromal amount within tumor, nuclear grade, nucleolar grade, presence of microvascular invasion, micro/macrosatellites, and presence of multiple nodules).

## 3 Results

### 3.1 Correlation between snoRNA expression clinical and pathological variables

The examination of continuous variables was carried out using Spearman correlation analysis. Notably, the analysis revealed a positive correlation (0.48) between the expression of snoRA80E and AFP, as presented in (Table 2).

**Table 2:** Correlation with continuous and ordinal variables.

Variables	snoRNA −12		snoRNA-47		snoRNA −80E		snoRNA −126	
	rho	P-val	rho	P-val	rho	P-val	rho	P-val
Age	0.03	0.85	0.11	0.56	0.04	0.83	−0.03	0.85
Sex	−0.02	0.92	0.18	0.33	0.08	0.65	0.19	0.29
Tumor grade	−0.05	0.77	−0.05	0.8	−0.04	0.82	−0.09	0.62
Tumor size	0.04	0.83	0.03	0.89	0.04	0.84	−0.01	0.95
Growth pattern	−0.09	0.72	0.02	0.93	−0.08	0.75	−0.2	0.41
Grade of fibrous encapsulation	−0.05	0.79	−0.14	0.44	−0.06	0.72	0.14	0.43
Architectural grade	−0.23	0.2	−0.2	0.27	−0.16	0.38	−0.27	0.12
Amount of stroma within the tumor	0.04	0.84	−0.03	0.89	−0.04	0.85	−0.14	0.44
Nuclear grade	0	0.99	0	0.98	−0.03	0.87	0.01	0.94
Nucleolar grade	0.13	0.46	0.2	0.29	0.2	0.26	0.08	0.66
Microvascular invasion	0.17	0.34	0.21	0.27	0.12	0.51	0.19	0.28
Micronodularity	0.13	0.47	0.13	0.48	0.17	0.33	0.17	0.34
Micro/macrosatelits	0.05	0.8	0.21	0.28	0.24	0.2	0.22	0.23
TNM rank	0.28	0.1	0.1	0.59	0.25	0.16	−0.09	0.63
AFP	0.16	0.47	0.25	0.29	**0.48**	**0.02**	0.13	0.54
Diabetes	−0.14	0.45	−0.16	0.39	−0.2	0.25	0.24	0.17

Bold value represents statistically significant.

The Mann-Whitney *U* test was applied for binary variables including gender, presence of microvascular invasion, presence of micro/macrosatellites, micronodularity, and amount of stroma within the tumor. Among these binary variables, no significant associations were observed. The Kruskal-Wallis test was used to analyze the association with growth pattern; however, no significant association was observed with the growth pattern.

### 3.2 The prognostic significance of snoRNAs

**Fig. 2:**
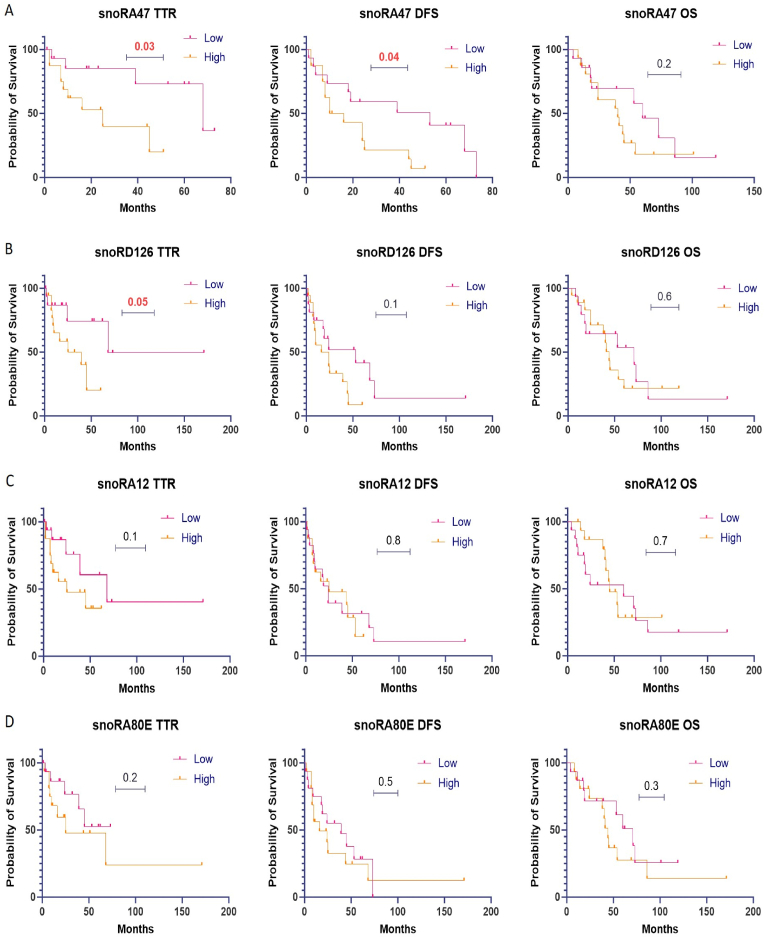
Survival Analysis: Fig. 2A, B, 2C, and 2D depict the survival curves for snoRA47, snoRD126, snoRA12, and snoRA80E, respectively. These curves represent the Time to Recurrence (TTR), Disease-Free Survival (DFS), and Overall Survival (OS) outcomes. The significance level (P < 0.05) indicates statistically significant differences in survival probabilities among the groups analyzed. Also, significant results were highlighted.

We classified expression levels as either high or low based on their median value. Values above the median indicate high expression, whereas values below the median indicate low expression. Our findings demonstrate that low expression of snoRA47 and snoRD126 was associated with better patient outcomes. Specifically, we observed that low expression of snoRA47 was associated with longer TTR (p = 0.03) and DFS (p = 0.04). Similarly, low expression of snoRD126 was notably associated with longer TTR (p = 0.05). However, it's important to note that snoRD126 displayed significance solely in its association with TTR but was not significant for DFS and OS, as illustrated in (Fig. 2A for snoRA47 and Fig. 2B for snoRD126). On the contrary, snoRA80E and snoRA12 did not exhibit any association with the patient outcomes TTR, DFS and OS based on our analysis (Fig. 2C for snoRA12 and Fig. 2D for snoRD80E).

### 3.3 Combined expression values and their prognostic value

In addition, we performed a combination analysis and classified snoRNA's expression levels correspondingly. When both snoRNA expression levels were low, we categorized them as low expression. When one snoRNA expression level was low and the other was high, or conversely, we considered them as medium expression. When both snoRNA expression levels were high, we interpreted them as high expression.

**Fig. 3:**
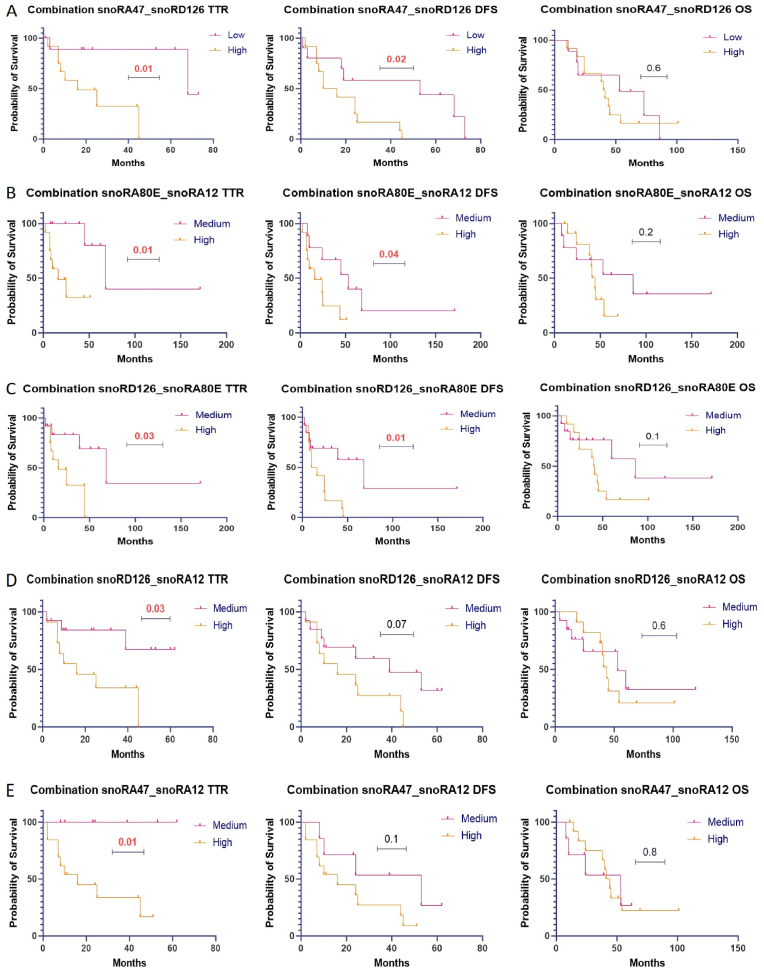
The combination analysis of snoRNAs in survival reveals significant associations between specific pairs across various figures (Fig. 3A: snoRA47_snoRD126, Fig. 3B: sno80E_snoRA12, Fig. 3C: snoRD126_snoRA80E, Fig. 3D: snoRD126_snoRA12, Fig. 3E: snoRA47_snoRA12), all indicating p-values significantly less than 0.05. These combinations demonstrate potential cooperative or synergistic effects on key survival metrics including time to recurrence (TTR), disease-free survival (DFS), and overall survival (OS). These findings underscore the importance of considering the collective impact of multiple snoRNAs in prognostic assessments and highlight potential avenues for further investigation in cancer management and therapeutic targeting.

We observed that combining low expressions of snoRA47 and snoRD126 was significantly associated with longer TTR and DFS (p = 0.01 and p = 0.02 respectively) but not with OS (Fig. 3A). Additionally, the combined medium expression levels of snoRA80E and snoRA12 were associated with longer TTR and DFS (p = 0.01 and p = 0.04 respectively) (Fig. 3B). The medium expression levels of snoRD126 and snoRA80E were associated with longer TTR and DFS (p = 0.03 and p = 0.01 respectively), as shown (Fig. 3C). The combination medium expression levels of snoRD126 and snoRA12 were associated with longer TTR (p = 0.03) (Fig. 3D). This significant association was observed only for TTR, but not DFS, as depicted in (Fig. 3D). Also, the combined medium expression levels of snoRA47 and snoRA12 were significantly associated with longer TTR (p = 0.01), but not with DFS and OS as shown in (Fig. 3E). We did not observe any significant association with OS in our cohort because our sample size is very low.

No significant outcomes were associated with combinations of low, high, or medium expression levels. This includes combinations involving snoRA47 and their impact on TTR, DFS, and OS (Fig. 4A). Similarly, no significant associations were found between the combined low, medium, or high expression levels of snoRA80E and snoRA12, snoRD126 and snoRA80E, snoRD126 and snoRA12, and TTR, DFS, and OS, as shown in Supplementary 1 (Fig. 4B–E).

## 4 Discussion

The investigation into the distinct roles of specific snoRNAs and their collective impact holds significant promise for advancing the accuracy of prognostic markers, enhancing the prediction of patient outcomes such as TTR and DFS in non-viral HCC. Our study, for the first time, delves into the expression patterns of snoRNAs within the unique context of non-viral HCC. One notable finding of our research is the correlation between the low expression level of snoRA47 and extended TTR and DFS, underscoring its potential as a prognostic indicator with clinical significance. Conversely, we observed upregulation of snoRA80E and a positive correlation between snoRA80E and AFP, indicating a potential link to adverse clinical outcomes.

In our study, we observed an upregulation of snoRA80E, also known as SNORA42, Box H/ACA 42, and ACA42 [19]. It is well known to play an important role in various cancers including CRC and HCC [13]. In another study on snoRA42 expression was upregulated in tissue and serum samples, positively associated with OS in oesophageal squamous carcinoma cells [20]. According to the literature, snoRA47 has the potential to serve as a predictive marker for HCC development. Wang G et al. confirmed snoRA47 overexpression in HCC tissues and cell lines. The overexpression of snoRA47 has been associated with cell proliferation, migration, and apoptosis, suggesting its potential role as a prognostic biomarker for HCC [13]. Li G et al.'s observation of snoRA47's overexpression in HCC tissues compared to normal tissues aligns with previous research. The Kaplan-Meier analysis performed by Li G et al. indicates that high snoRA47 expression is associated with shorter OS. Whereas, in our study snoRA47 was upregulated, and we observed that the low expression of snoRA47 was associated with longer TTR and DFS but not with OS. This suggests that snoRA47 might have a more specific influence on certain aspects of HCC progression, or that additional factors may be influencing OS outcomes [16]. The finding of Xu W et al. [18] revealed that high expression of snoRD126 was associated with shorter OS. Within a small cohort of our study, a similar effect was observed concerning patient survival for TTR but not OS.

Our proposal to combine snoRD126 and snoRA80E shows potential in prognostication. The combination of snoRD126 and snoRA80E, with medium expression was associated with longer TTR and DFS. These observations suggest that snoRD126 might have an impact on different patient outcomes in non-viral HCC, and when combined with snoRN80E, it may exhibit a notable association with TTR and DFS. These results emphasize the combined effects of specific combinations of snoRNA expressions on patient outcomes. Despite the limitations, there is a strong conviction that conducting a follow-up study with a larger number of cases and exploring a broader range of snoRNA alterations could significantly enhance and refine the prognostic tools established in the current study. The additional data obtained from a more extensive cohort could prove invaluable in refining treatment decision-making processes, offering a more comprehensive understanding of the prognostic significance of various snoRNA alterations in HCC. These findings underscore the potential prognostic significance of snoRA47 and snoRD126 in predicting survival outcomes, particularly in relation to time to recurrence while highlighting the limited impact of snoRA80E and snoRA12 in this context.

## 5 Conclusion

The implications of this research could be far-reaching, potentially influencing clinical strategies for prognosis assessment and treatment planning in non-viral related HCC. If validated, these findings might contribute significantly to personalized medicine and patient care within this specific subset of HCC. These findings regarding molecular markers in cancer prognosis underscore the necessity for additional investigation and validation in larger cohorts examining the role of 4 snoRNAs as potential prognostic markers in non-viral HCC.

## Ethics approval and consent to participant

The Ethical approval for the study was given by the Ethical Board of the Faculty of Medicine and University Hospital in Pilsen (114/2022, April 07, 2022). The need for informed consent was waived by the Ethical Board of Faculty of Medicine and University hospital in Pilsen (114/2022, April 07, 2022) because of the retrospective nature of the study. This study was carried out in accordance with the ethical standards laid down in the Declaration of Helsinki (2013 version).

## Funding

This research has received funding from the grants from the 10.13039/501100003243Ministry of Health of the Czech Republic AZV NU21–03-00506, and by the project 10.13039/100012928National Institute for Cancer Research—NICR (Programme EXCELES, ID Project No. LX22NPO5102), funded by the 10.13039/501100003529European Union—Next Generation EU, Grant Agency of Czech Republic 23-05609S.

## Conflicts of interest

All authors have read and agreed to the published version of the manuscript.

## CRediT authorship contribution statement

**Venkata Ramana Mallela:** Writing – original draft, Methodology, Investigation, Formal analysis, Data curation, Conceptualization. **Phanindra Babu Kasi:** Conceptualization, Data curation, Formal analysis, Methodology, Validation, Writing – original draft, Writing – review & editing, Writing – review & editing, Writing – original draft, Visualization, Validation, Project administration, Methodology, Investigation, Formal analysis, Data curation, Conceptualization. **Dattatrya Shetti:** Data curation, Formal analysis, Writing – review & editing, Methodology, Data curation. **Andriy Trailin:** Writing – review & editing, Data curation. **Lenka Cervenkova:** Writing – review & editing, Data curation. **Richard Palek:** Writing – review & editing, Data curation. **Ondřej Daum:** Writing – review & editing, Data curation. **Vaclav Liska:** Writing – review & editing, Supervision, Project administration, Funding acquisition, Data curation. **Kari Hemminki:** Writing – review & editing, Supervision, Project administration, Funding acquisition. **Filip Ambrozkiewicz:** Writing – review & editing, Supervision, Project administration, Investigation.

## List of abbreviations

HCC: hepatocellular carcinoma
SnoRNA: small nuclear RNA
HBV: hepatitis B Virus
HCV: hepatitis C Virus
NASH: non-alcoholic steatohepatitis
NAFLD: non-alcoholic fatty liver diseases
NTAT: non-tumor adjacent tissue
TTR: Time to recurrence
DFS: Disease-free survival
OS: Overall survival
