# Supplementary material for: Small nucleolar RNA expression profiles: A potential prognostic biomarker for non-viral Hepatocellular carcinoma
Source: Noncoding RNA Res. 2024 Jun 12;9(4):1133–9. doi: 10.1016/j.ncrna.2024.06.009 (PMC11254499; doi:10.1016/j.ncrna.2024.06.009)

**Small Nucleolar RNA Expression Profiles: A Potential Prognostic Biomarker for Non-Viral Hepatocellular Carcinoma (Supplementary 1).**


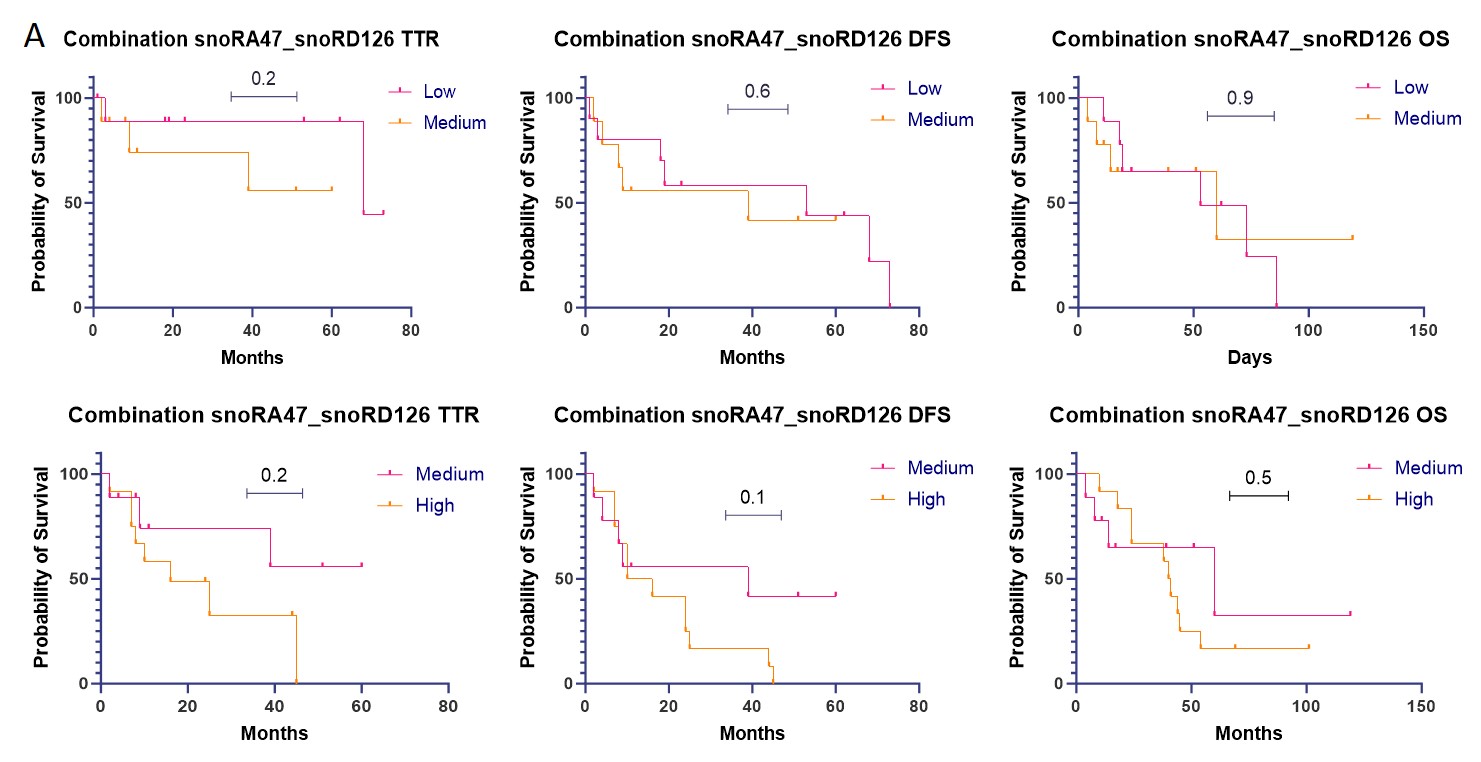

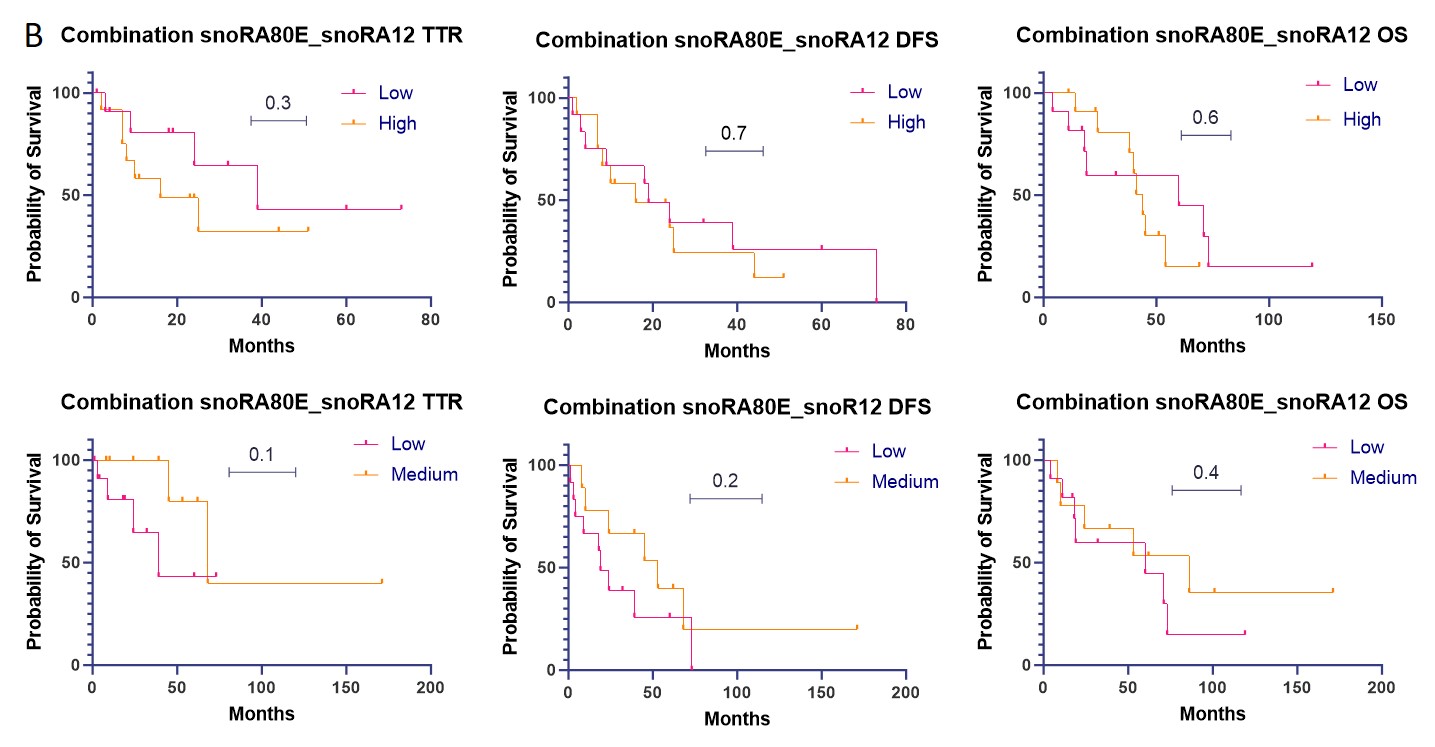

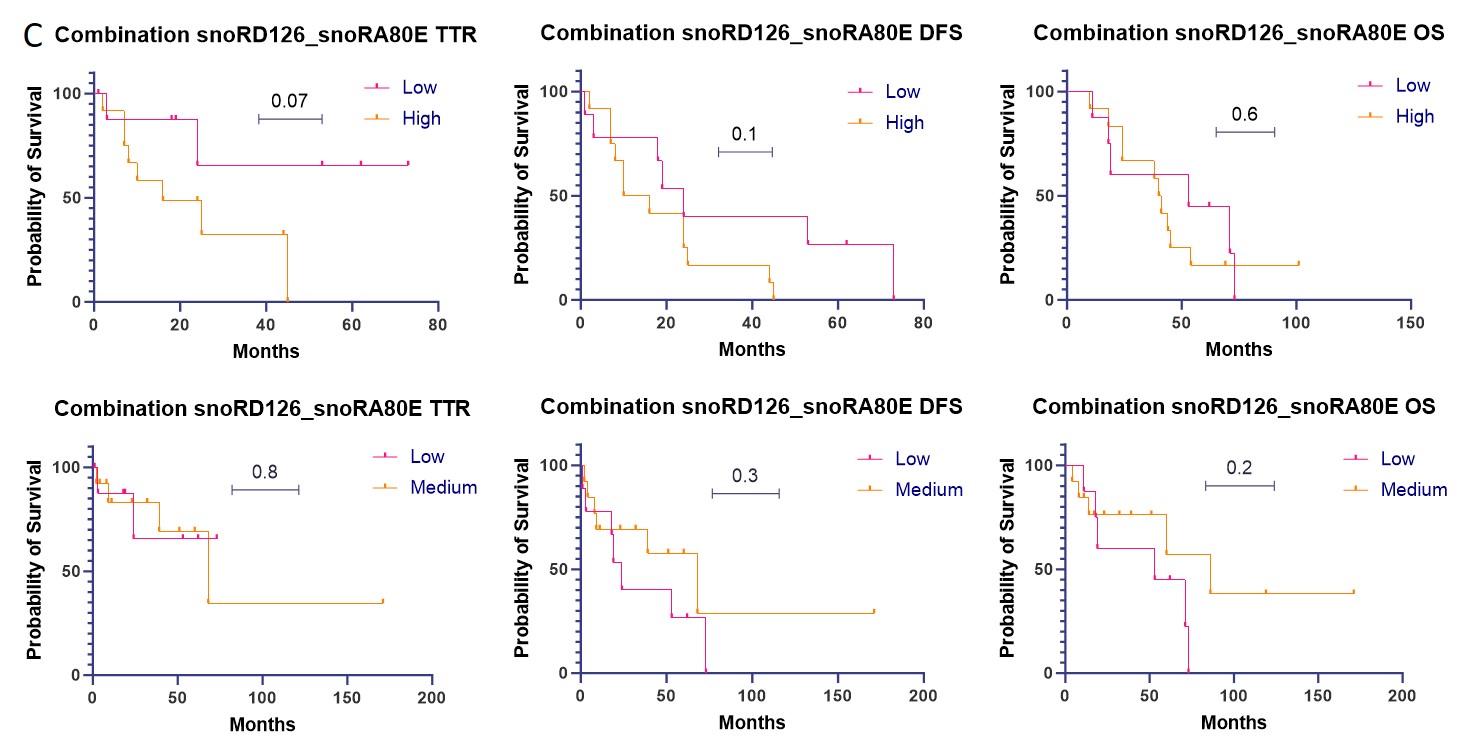

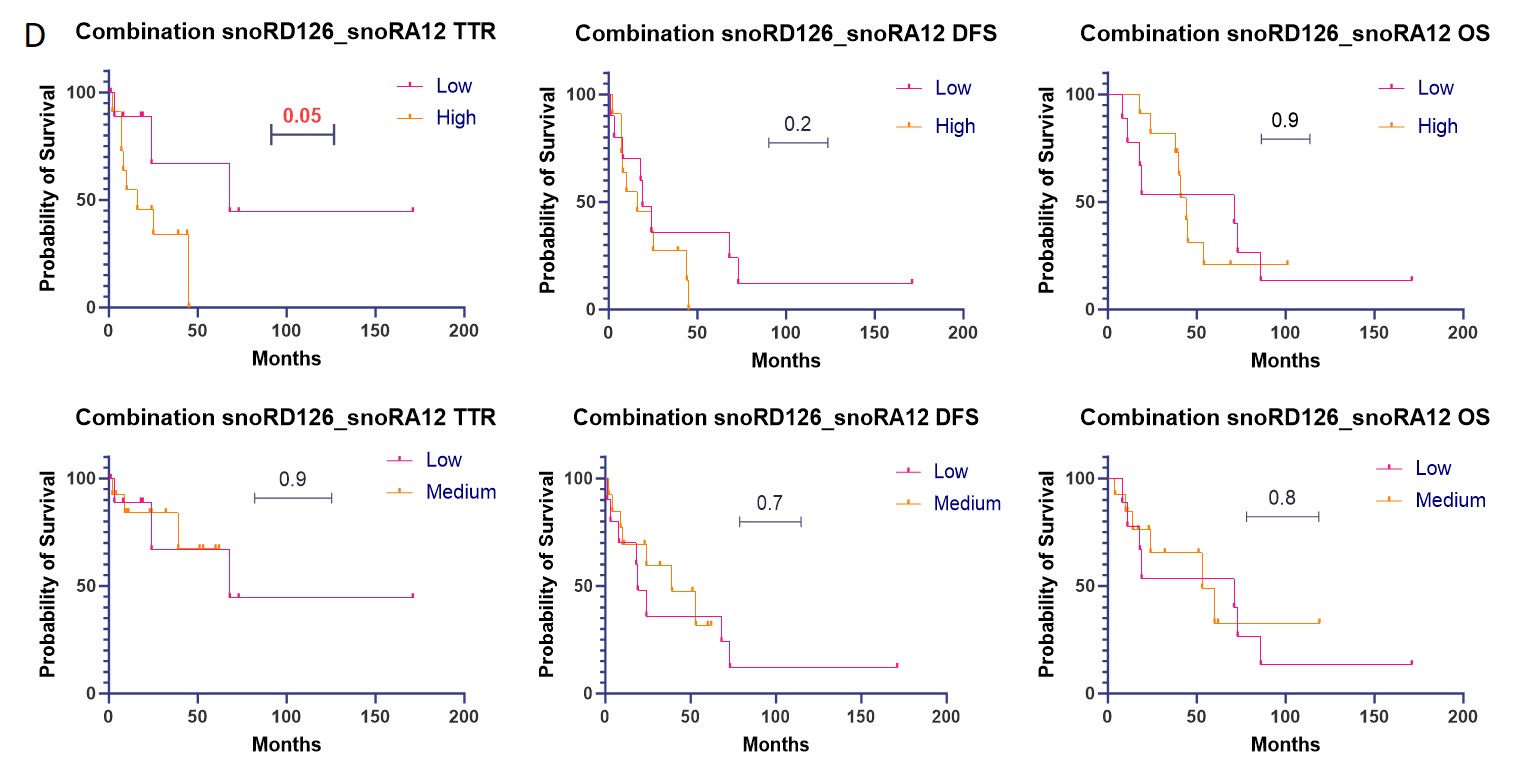


Figure 4. The combination of low or high, low or medium expressions was not associated with any significant outcome. snoRA47 and TTR, DFS, and OS (Fig. 4A). Similarly, we did not find any significant associations between the combined low or medium expressions of snoRA80E and snoRA12, snoRD126 and snoRA80E, snoRD126 and snoRA12, with TTR, DFS, and OS, as shown in Supplementary 1 (Figures 4B–4E).
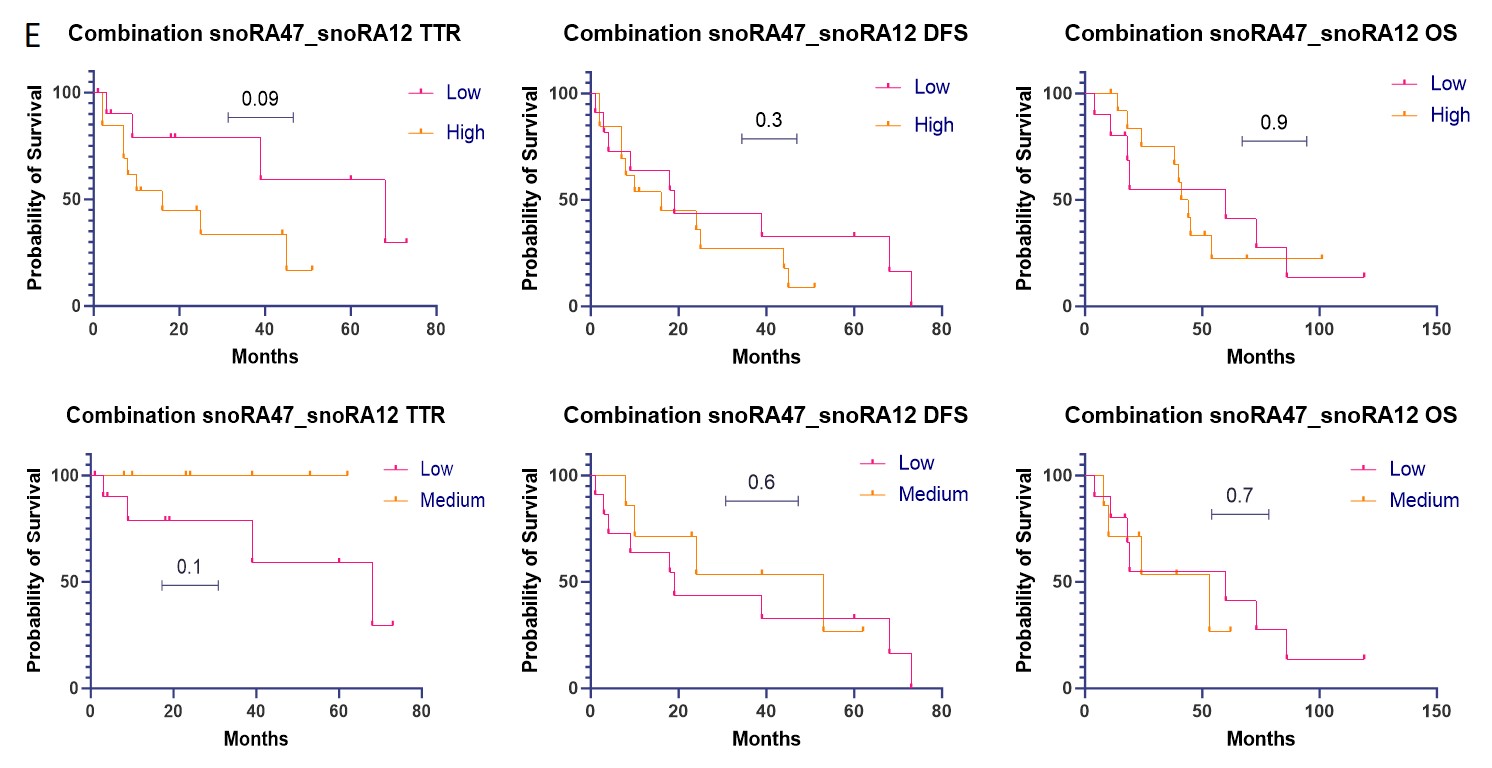

Supplement: Multimedia component 1 [file mmc1.docx]
